# The Story of the Insane from Year to Year

**Published:** 1894-10-20

**Authors:** 


### THE STORY OF THE INSANE FROM YEAR TO YEAR.

(7th Series.)

### THE DERBY COUNTY ASYLUM AT MICKLEOVER.

Here the numbers fell from 460 to 450; but unfortunately this decrease was not owing to any diminution in the number of Derbyshire lunatics, but to the crowded state of the asylum, necessitating the transfer of 16 patients to the Nottingham Asylum. There were 127 admissions, 49 recoveries, and 43 deaths. Thrown into percentages in the usual way in asylums, we find the recoveries coming out above the average, at 42·2, and the deaths a little below the average, at 9·3. Hereditary influence accounts for 36 of the admissions, and drink for 12. Sixteen of the deaths were caused by cerebral or spinal diseases, 10 by consumption, and one by dysentery. The training of attendants and nurses has been continued, with the result that twelve of the staff passed the examination of the Medico-Psychological Association. Maria Dawson, the head laundress, is to be congratulated on having obtained her pension, and it is to be hoped she will long live to enjoy it. A pension scheme has been drawn up and approved, and, on the date of the report, was awaiting the approval of the County Council. This will make the fourth county which has accepted a definite scheme for pensioning asylum officials, and it is much to be desired the ball will be kept rolling until every asylum has adopted this or some similar scheme. The asylum is being added to, and a new residence is being built for the medical superintendent. The weekly rate of maintenance is 10s. 4½d. per head.

### WILTS COUNTY ASYLUM, DEVIZES.

When the year 1893 closed there were in this asylum 710 patients, being an increase of 17 since January 1st. There were 135 admissions, 49 recoveries, and 48 deaths. The recoveries are at the percentage of 38·2 on the admissions, and the deaths at 6·7 on the average number resident. Hereditary tendency claims 53 of the year's admissions, and drink accounts for 9, while 21 were driven insane by domestic trouble, and 10 by religious excitement. As usual the chief cause of death was some form of brain disease, and consumption killed 4. The asylum is an old one, and there is not an accurate balance between the day and night spaces. Mr. Bowes recommends certain alterations which would do away with this discrepancy and at the same time provide proper sick dormitories. It is well known that the asylum has immensely improved under Mr. Bowes' superintendentship, and the present report shows that it is still marching onwards. The sewage arrangements have been entirely remodelled, new locks have been fitted throughout, electric bells have been provided, and additional land has been added to the estate. Mr. Bowes thinks the word "Asylum" should give place to that of "Hospital," the latter being more in harmony with the work done in the institution, and also more agreeable to the patients and their friends. We agree with Mr. Bowes, and we gladly quote his statement that "the word Asylum is associated with such horrors of the past, which necessarily lead to suspicions in the present day, and apart from this it has a depressing effect upon the patients, who are apt to look upon the institution as a place of of detention." Why not begin the new designation in the next annual report? And if Mr. Bowes will join to this innovation the disuse of locked doors by day, and the destruction of airing court walls, he will have done much to earn the gratitude of both patients and officials. The committee testify "to the careful and efficient manner in which the Asylum has been managed by the medical superintendent." The weekly rate is 8s. 11d. per head.

